# Early benefit assessment (EBA) in Germany: analysing decisions 18 months after introducing the new AMNOG legislation

**DOI:** 10.1007/s10198-013-0495-y

**Published:** 2013-06-16

**Authors:** Jörg Ruof, Friedrich Wilhelm Schwartz, J.-Matthias Schulenburg, Charalabos-Markos Dintsios

**Affiliations:** 1 Roche Pharma AG, Emil-Barrell-Str. 1, 79639 Grenzach-Wyhlen, Germany; 2 Medical School of Hanover, Hanover, Germany; 3 Leibniz University of Hanover, Hanover, Germany; 4 German Association of Research-based Pharmaceutical Companies (vfa), Berlin, Germany; 5 Department of Public Health, Heinrich Heine University, Düsseldorf, Germany

**Keywords:** Health care reform, (Early) benefit assessment, Appropriate comparative therapy, Market access, AMNOG, I10, I11, I18

## Abstract

**Objectives:**

Since the introduction of the German health care reform in January 2011, an early benefit assessment (EBA) is required for all new medicines. Pharmaceutical manufacturers have to submit a benefit dossier for evaluation by the Institute for Quality and Efficiency in Health Care (IQWiG). A final decision is made by the Federal Joint Committee (G-BA). The aim of this investigation was to analyse the outcomes 18 months after introduction of the new legislation and to identify critical areas requiring further discussion and development.

**Methods:**

All EBAs commenced prior to June 2012 were included. The G-BA website was used to obtain manufacturers’ benefit dossiers, IQWiG assessments, and G-BA decisions. Four areas of interest were analysed: levels of additional benefit, appropriate comparative therapy (ACT), patient-relevant endpoints, and adverse events.

**Results:**

Twenty-seven EBAs were analysed. IQWiG stated a benefit in 50 % of EBAs, whereas G-BA stated a benefit in 63 %, but only in 50 % of identified subgroups and 40 % of patients involved. In 12 EBAs, the ACT suggested by G-BA differed from the comparator used in phase III trials. The G-BA reported no benefits on health-related quality of life. Discrepancies arose in morbidity outcomes such as ‘progression-free survival’ and ‘sustained virological response’. Categorisation and balancing of adverse events was conducted within various assessments.

**Conclusions:**

Considerable variance was observed in the levels of additional benefit reported by pharmaceutical manufacturers, IQWiG and G-BA. The areas of disagreement included ACT selection, definition of subgroups and patient-relevant endpoints, and classification and balancing of adverse events.

## Introduction

**Fig. 1:**
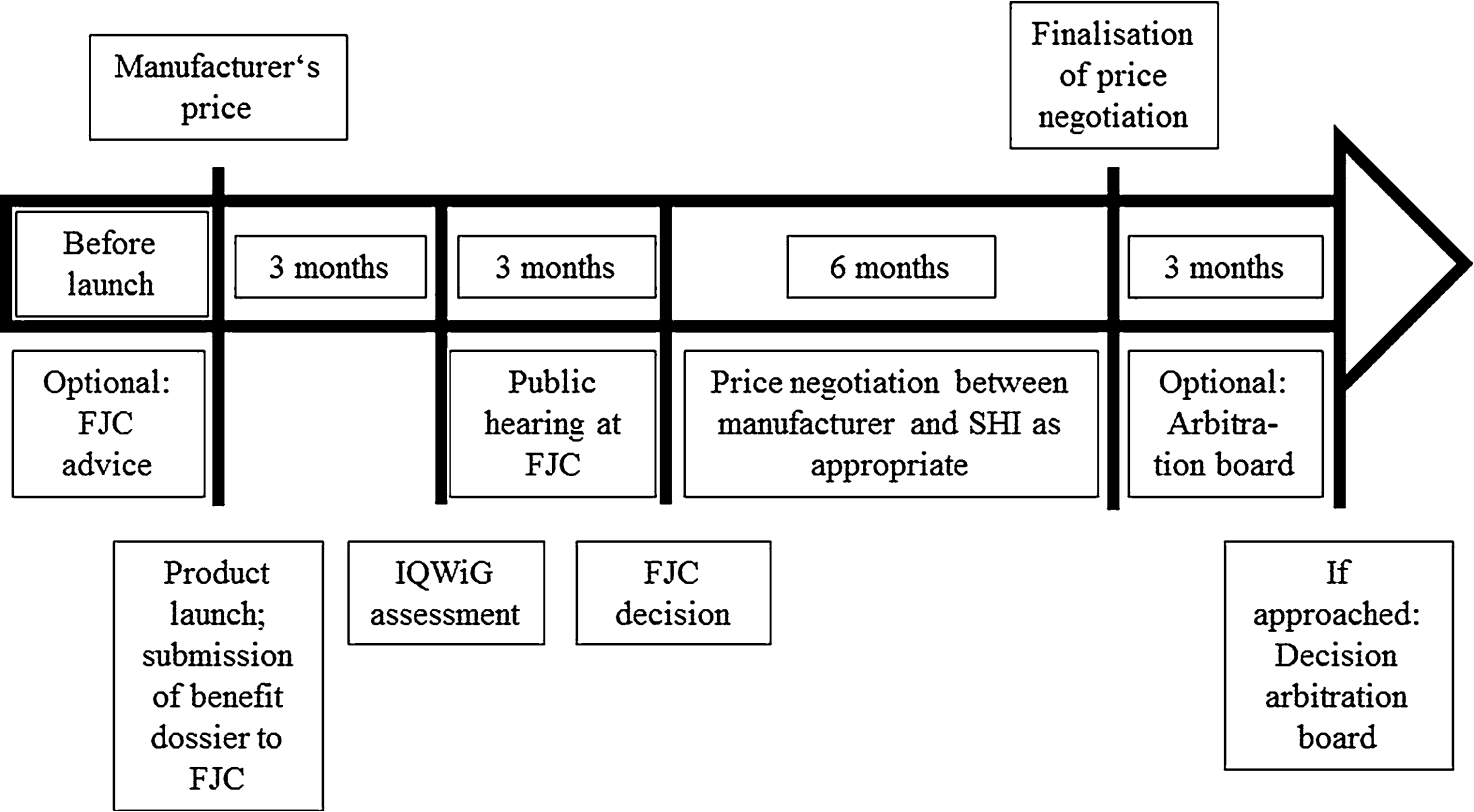
Flow chart covering benefit assessment and price negotiation according to the new German regulations since January 2011

The new Act to Reorganize the Pharmaceuticals Market in the Statutory Health Insurance (SHI) System [Gesetz zur Neuordnung des Arzneimittelmarktes in der gesetzlichen Krankenversicherung (AMNOG)] [1], which was introduced by the German Parliament based on an initiative of the Ministry of Health, passed through Federal Parliament on 11 November 2010 and came into effect on 1 January 2011. A key component of AMNOG is the introduction of a mandatory benefit assessment, with the subsequent price negotiation process for new medicines to be completed within 1 year of product launch (Fig. 1) [1]. Pharmaceutical manufacturers have to submit a benefit dossier to the Federal Joint Committee (Gemeinsamer Bundesausschuss, G-BA), the key legal institution of the self-administration within the German health care system, before the medicine is made commercially available in Germany. The G-BA is the highest decision-making body of the joint self-governing board of stakeholders in healthcare (physicians, dentists, hospitals and health insurance funds) in Germany. The manufacturer may request an advice meeting with the G-BA in order to determine the appropriate comparative therapy (ACT) and address any other relevant questions. Within 3 months of submission, the dossier is evaluated in most cases by the Institute for Quality and Efficiency in Health Care (IQWiG) [2]. The IQWiG evaluation results in a recommendation regarding the additional patient-relevant benefit of the investigated drug. Three months after IQWiG’s recommendation, the G-BA concludes the benefit assessment by making a final decision regarding the additional benefit. The G-BA decision is based on the manufacturers’ dossier, IQWiG evaluation, as well as the results of a public hearing. After the G-BA decision, price negotiations between the SHI and the manufacturer begin. The price negotiations must be finalised within 6 months. If no agreement is reached in this time, an arbitration board is called, which must reach a final pricing decision within 3 months.

Various key elements may be discriminated within the AMNOG process [1, 3]. An ACT is used to determine the additional benefit of the new medication. The ACT should be identified based on the standards of evidence-based medicine, the contents of the marketing authorisation, recommendations in treatment guidelines, and other criteria [1, 3]. The level of additional benefit versus the ACT is categorised as: (1) major; (2) significant; (3) marginal; (4) not quantifiable; (5) no; or (6) less.

The methodological basis of the benefit assessment and uncertainties regarding outcome and study results are covered in IQWiG’s publication on ‘General Methods’ [4]. Accordingly, the evidence base is grouped into the categories of ‘proof’, ‘indication’, or ‘hint’ based upon the number and characteristics of studies provided, the certainty of results, and the observed effects (Table 1) [4].

**Table 1:** Requirements for the assessment of level of evidence for an additional benefit [4]

Conclusion	Requirement		
	Number of studies	Certainty of results	Effect
Proof	≥2	Mostly high	In the same direction
Indication	≥2	Mostly moderate	In the same direction
	1	High	Statistically significant
Hint	≥2	Mostly low	In the same direction
	1	Moderate	Statistically significant

Outcomes considered by IQWiG and G-BA in terms of additional benefit are grouped into three dimensions: mortality, morbidity and health-related quality of life (HRQoL) [3, 4]. Fewer adverse events compared with the ACT is considered an additional benefit of the new medicine. All available information on adverse events has to be included in the dossier [3, 4].

So far, only very few new products have passed through the full AMNOG process, including price negotiations. However, a considerable amount of experience has been gathered regarding the early part of the process, i.e. the early benefit assessment (EBA). The aim of our investigation was to analyse the outcomes 18 month after introducing the new legislation and to identify critical areas that require further discussion and development.

## Methods

This analysis included all EBAs that commenced prior to 1 June 2012. The G-BA website (http://www.g-ba.de/informationen/nutzenbewertung) was used to obtain the manufacturers’ benefit dossiers, the IQWiG assessments, and the G-BA decisions. The analysis specifically addressed four areas of interest.

1. Levels of additional benefit as stated by the IQWiG and G-BA were compared. Positive (category 1–4: major; significant; marginal; not quantifiable additional benefit) and negative (category 5–6: no/less additional benefit) decisions were discriminated. Subgroup analyses (‘slicing’) conducted by IQWiG and G-BA were reviewed. Both total benefit scores and subgroup scores were included in the analysis. To compare total scores from IQWiG and G-BA, either the total score (if provided) or the best available subgroup score was used. Discrepancies between IQWiG and G-BA decisions were identified and analysed. Levels of evidence (proof, indication, hint) reported by IQWiG and G-BA were compared.
2. The ACT suggested by the G-BA was compared with the ACT within the manufacturers’ dossier. Furthermore, phase III comparators were included, as derived from the European Public Assessment Reports (EPARs), which were downloaded from the website of the European Medicines Agency (EMA) [5].
3. Additional benefits were categorised according to the three dimensions stated within the relevant social law, i.e. §35 (1b) of the German Social Code Book V [6]: mortality, morbidity, HRQoL. The manufacturers’ dossiers were reviewed regarding their respective claims. Decisions by IQWiG and G-BA were analysed and compared regarding their acceptance of those claims.
4. Institute for Quality and Efficiency in Health Care and G-BA decisions regarding adverse events, as well as the weighting applied when deriving a total score, were analysed.

## Results

Thirty-one EBAs commenced prior to 1 June 2012 (Table 2). The G-BA exempted ceftaroline fosamil, dexmedetomidine, and piperaquine tetraphosphate/dihydroartemisinin from an EBA [7] due to low expected costs for the SHI. Additionally, the assessment of olmesartan medoxomil/amlodipine/hydrochlorothiazide was discontinued by the G-BA [7]. Therefore, these four products were not included in this analysis, resulting in inclusion of 27 EBAs.

**Table 2:** New medicines in the EBA process

Drug	Brand name	Indication	Manufacturer	Start date EBA
Abiraterone acetate	Zytiga^®^	Prostate cancer	Janssen-Cilag	01.10.2011
Aliskiren/amlodipine	Rasilamlo^®^	Hypertension	Novartis	15.05.2011
Apixaban	Eliquis^®^	Prophylaxis of venous thromboembolism after athroplasty (hip or knee replacement)	Bristol-Myers Squibb	15.06.2011
Azilsartan medoxomil	Edarbi^®^	Hypertension	Takeda	15.01.2012
Belatacept	Nulojix^®^	Graft rejection Kidney transplantation	Bristol-Myers Squibb	15.07.2011
Belimumab	Benlysta^®^	Systemic lupus erythematosus	GlaxoSmithKline	27.07.2011
Boceprevir	Victrelis^®^	Chronic hepatitis C	MSD Sharp & Dohme	01.09.2011
Bromfenac	Yellox^®^	Inflammation in the eye following operation to remove cataract	Bausch und Lomb/Dr. Mann	01.08.2011
Cabazitaxel	Jevtana^®^	Prostate cancer	Sanofi-Aventis	15.04.2011
Ceftaroline fosamil	Zinforo^®^	Skin and soft-tissue infections, community-acquired pneumonia	AstraZeneca	14.03.2012
Dexmedetomidine	Dexdor^®^	Conscious sedation	Orion	13.07.2011
Emtricitabine/rilpivirine/tenofovir disoproxil	Eviplera^®^	HIV infection	Gilead	15.01.2012
Eribulin	Halaven^®^	Breast cancer	Eisai	01.05.2011
Extract of *Cannabis sativa*	Sativex^®^	Spasticity in multiple sclerosis	Almirall Hermal	01.07.2011
Fampridine	Fampyra^®^	Multiple sclerosis	Biogen Idec	29.07.2011
Fingolimod	Gilenya^®^	Multiple sclerosis	Novartis	15.04.2011
Ipilimumab	Yervoy^®^	Melanoma	Bristol-Myers Squibb	01.08.2011
Linagliptin	Trajenta^®^	Diabetes mellitus type II	Boehringer Ingelheim	01.10.2011^a^
Microbial collagenase	Xiapex^®^	Dupuytren’s contracture	Pfizer	01.05.2011
Olmesartan medoxomil/amlodipine/hydrochlorothiazide	Sevikar HCT^®^	Hypertension	Daiichi Sankyo	No status
Piperaquine tetraphosphate/dihydroartemisinin	Eurartesim^®^	Malaria	Sigma-tau Arzneimittel	21.03.2012
Pirfenidone	Esbriet^®^	Idiopathic pulmonary fibrosis	InterMune	15.09.2011
Pitavastatin	Livazo^®^	Primary hypercholesterolemia and mixed dyslipidemia	Merckle Recordati	01.06.2011
Regadenoson	Rapiscan^®^	Myocardial perfusion record	Rapidscan Pharma Solutions	15.04.2011
Retigabine	Trobalt^®^^®^	Epilepsy (add-on)	GlaxoSmithKline	15.05.2011
Rilpivirine	Edurant^®^	HIV infection	Janssen-Cilag	15.01.2012
Tafamidis meglumine	Vyndaqel^®^	Amyloidosis	Pfizer	15.12.2011
Telaprevir	Incivo^®^	Hepatitis C	Janssen-Cilag	15.10.2011
Ticagrelor	Brilique^®^	Acute coronary syndrome	AstraZeneca	01.01.2011
Vandetanib	Caprelsa^®^	Thyroid neoplasms	AstraZeneca	15.03.2012
Vemurafenib	Zelboraf^®^	Melanoma	Roche	15.03.2012

^a^Re-assessment according to §35a (5b) German Social Code Book V had started on 01.09.2012

For four new drugs (azilsartan medoxomil, bromfenac, pitavastatin, regadenoson), the manufacturers did not submit a dossier, leading to a no additional benefit decision by the G-BA without an IQWiG evaluation [7]. For orphan drugs, market authorisation is considered proof of additional benefit by German regulations [§35a (1) German Social Code Book V], but only up to an annual revenue of 50 million Euros. Once this sales threshold is exceeded, orphan drugs are assessed as conventional drugs [6]. Therefore, pirfenidone and tafamidis meglumine were only investigated in terms of their level of additional benefit, and not for the level of proof.

### Additional benefit

**Fig. 2:**
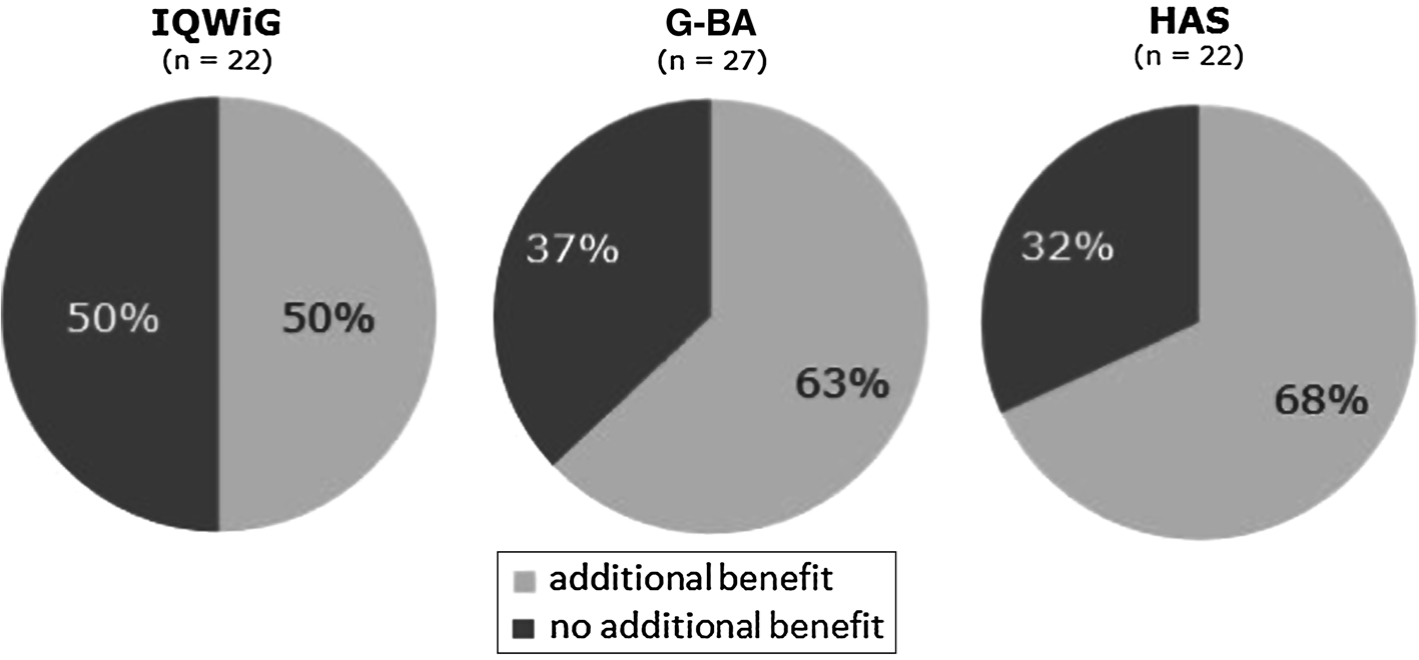
Presence of additional benefit as reported by IQWiG, G-BA and HAS according to the number of products evaluated (*n*)

Table 3 summarises the recommendations by IQWiG and the respective G-BA decisions, in terms of additional benefit, for the 27 products considered here. IQWiG concluded that there were significant and marginal additional benefits in six and three EBAs, respectively, and a non-quantifiable additional benefit was concluded in two assessments. Half of the new medicines were rated as having no additional benefit by IQWiG (Fig. 2) [7]. Overall, G-BA decisions were more positive than the IQWiG recommendations, with G-BA concluding that about two-thirds of products had an additional benefit (Fig. 2) [7]. Important differences in the overall additional benefit between IQWiG recommendations and G-BA decisions were found in several EBAs [7].

**Table 3:** Comparison of IQWiG assessment and G-BA decision for new medicines regarding presence of additional benefit

	IQWiG	G-BA
Abiraterone acetate	+	+
Aliskiren/amlodipine	−	−
Apixaban	+	+
Azilsartan medoxomil	n.d.	−
Belatacept	+	+
Belimumab	−	+
Boceprevir	+	+
Bromfenac	n.d.	−
Cabazitaxel	+	+
Emtricitabine/rilpivirine/tenofovir disoproxil	−	+
Eribulin	−	+
Extract of *Cannabis sativa*	−	+
Fampridine	−	−
Fingolimod	+	+
Ipilimumab	+	+
Linagliptin	−	−
Microbial collagenase	−	−
Pirfenidone	−	+
Pitavastatin	n.d.	−
Regadenoson	n.d.	−
Retigabine	−	−
Rilpivirine	+	+
Tafamidis meglumine	n.d.	+
Telaprevir	+	+
Ticagrelor	+	+
Vandetanib	−	−
Vemurafenib	+	+

In case of different subgroups within an EBA, the best subgroup assessment was used

Information on http://www.g-ba.de/informationen/nutzenbewertung/ in manufacturers’ dossier and G-BA decision

+, additional benefit; −, no additional benefit; n.d., not determined

A hearing is established in between the time of recommendation by IQWiG and the time of the final decision by G-BA. The results of this hearing have an influence on the G-BA decision and could be considered as a reason for differences in the assessment of IQWiG and G-BA. However, the way the results of the hearing affect the final G-BA decision still remains unclear, because the respective G-BA subcommittee for drugs decides in closed meetings and the relationship between hearings and G-BA decisions is not easy to understand.

#### Belimumab

Institute for Quality and Efficiency in Health Care concluded no additional benefit, whereas G-BA concluded there was a significant additional benefit. The reason for the discrepancy was that G-BA accepted evidence versus the comparator used in the phase III trials while IQWiG disagreed with the ACT due to lack of adaptation of steroid dose within those trials.

#### Cabazitaxel

Institute for Quality and Efficiency in Health Care suggested a major additional benefit in terms of efficacy. However, because of the reported adverse events, they recommended a total score of significant additional benefit. G-BA also reduced the total additional benefit score by one level due to the side-effect profile. However, G-BA did not agree with IQWiG regarding the additional efficacy benefit and decided for a significant additional benefit in terms of efficacy and a total score of marginal additional benefit.

#### Emtricitabine/rilpivirine/tenofovir disoproxil

Institute for Quality and Efficiency in Health Care concluded there was no additional benefit. During the hearing process, the manufacturer submitted further clinical data. After evaluation of the new data G-BA concluded there was a marginal additional benefit. For rilpivirine monotherapy, IQWiG suggested a major additional benefit, whereas G-BA concluded a marginal additional benefit.

#### Eribulin

Institute for Quality and Efficiency in Health Care concluded there was no additional benefit in both patient populations, whereas G-BA concluded there was a marginal additional benefit in patients that cannot be re-exposed to anthracycline and/or taxane treatment and less benefit in patients that can be re-exposed to those treatments. The key reason for the discrepancy was different weighting of damage potential within the two subgroups between IQWiG and G-BA.

#### Extract of *Cannabis sativa*

Institute for Quality and Efficiency in Health Care suggested no additional benefit, whereas G-BA concluded a marginal additional benefit. While IQWiG suggested that the manufacturer did not match the suggested ACT, G-BA accepted the data provided by the manufacturer.

Study populations were divided into subgroups more frequently by IQWiG than by the manufacturers. In some cases G-BA disregarded the patient subgroups recommended by IQWiG and analysed different patient populations instead [7]. Importantly, the ACT defined by G-BA may vary for different subgroups.

In five assessments, comparative data versus ACT were missing for defined subgroups. IQWiG and G-BA were not able to identify any additional benefit for certain subgroups, i.e. in the EBAs for abiraterone acetate, cabazitaxel, fingolimod, microbial collagenase and ticagrelor [7].

For eribulin, only some of the data presented by the manufacturer were deemed relevant by IQWiG and G-BA for the specified subgroups, leading to a decrease in the level of evidence for additional benefit [7]. Omission of data for relevant populations led to the dossier for vandetanib being deemed unacceptable for benefit assessment by IQWiG and G-BA [7]. Similarly, IQWiG considered the dossier of emtricitabine/rilpivirine/tenofovir disoproxil as incomplete due to missing investigations regarding subgroup differences [7].

In six benefit decisions by G-BA a time limit was imposed.

### Appropriate comparative therapy (ACT)

Table 4 summarises comparators used in phase III clinical trials, the ACT used in the manufacturers’ dossiers, and the ACT as proposed by G-BA for products, where discrepancies exist.

**Table 4:** Comparison of ACT used by manufacturer in the EBA dossiers, ACT defined by G-BA, and comparators used in phase III trials

	Phase III comparator [5]	ACT in manufacturers’ dossier^a,b^	ACT defined by G-BA^a,b^
ACT recommendation by G-BA different from Phase III comparator but accepted by manufacturer			
Fampridine	Placebo (add-on immunomodulatory therapy)	Physiotherapy	Physiotherapy according to German remedies regulations; OST for multiple sclerosis
Abiraterone acetate^c^	Placebo (add-on to prednisone or prednisolone)	II: Docetaxel	II: Docetaxel (add-on to prednisone, prednisolone)
Fingolimod^d^	a) Placebo b) β-Interferon	I: Glatiramer acetate	I: Glatiramer acetate
Vandetanib	Placebo	BSC	BSC
ACT recommendation by G-BA not accepted by manufacturer			
Microbial collagenase^e^	Placebo	Partial fasciectomy (PF)	I: No therapy II: Percutaneous needle fasciotomy (PNF) III: PF IV: PNF
Ticagrelor^f^	Clopidogrel + ASA	Clopidogrel + ASA	III: Prasugrel + ASA IV: Monotherapy with ASA
Cabazitaxel	Mitoxantrone (add-on to prednisone and prednisolone)	Mitoxantrone (add-on to prednisone and prednisolone)	I (BSC): Dexamethasone, prednisone, prednisolone or methylprednisolone + BSC
Pirfenidone	Placebo	Not determined	BSC
Aliskiren/amlodipine	Aliskiren and amlodipine alone	Aliskiren and amlodipine alone	Combination of ACE-inhibitor (lisinoprile or ramiprile or enalaprile) and calcium-antagonist (amlodipine or nitrendipine)
Linagliptin^g^	Placebo alone or add on to metformin, a combination of metformin plus sulphonylurea or pioglitazone	Sitagliptin	I: Sulphonylurea (glibenclamide, glimepiride) II: Sulphonylurea (glibenclamide, glimepiride) + metformin III: Metformin + human insuline
Retigabine	Placebo	Lacosamide	Lamotrigine or topiramate

*ASA* acetylsalicylic acid, *BSC* best supportive care, *OST* optimised standard treatment

^a^Information on http://www.g-ba.de/informationen/nutzenbewertung/ in manufacturer’s dossier and G-BA decision

^b^ACT can differ among subgroups. Subgroups are marked with Roman numerals

^c^Patients, where re-exposure with Docetaxel is possible

^d^Relapsing-remitting multiple sclerosis (RRMS), non-responder to completed β-interferon therapy

^e^Subgroup classification according to disease severity (Tubiana stage)

^f^Subgroup classification according to indication (III: ST-elevation myocardial infarction (STEMI) managed with percutaneous coronary intervention (PCI); IV: STEMI managed with coronary artery bypass grafting (CABG)

^g^Subgroup classification according to use of mono- (I), dual (II) or triple (III) therapy with linagliptin

**Fig. 3:**
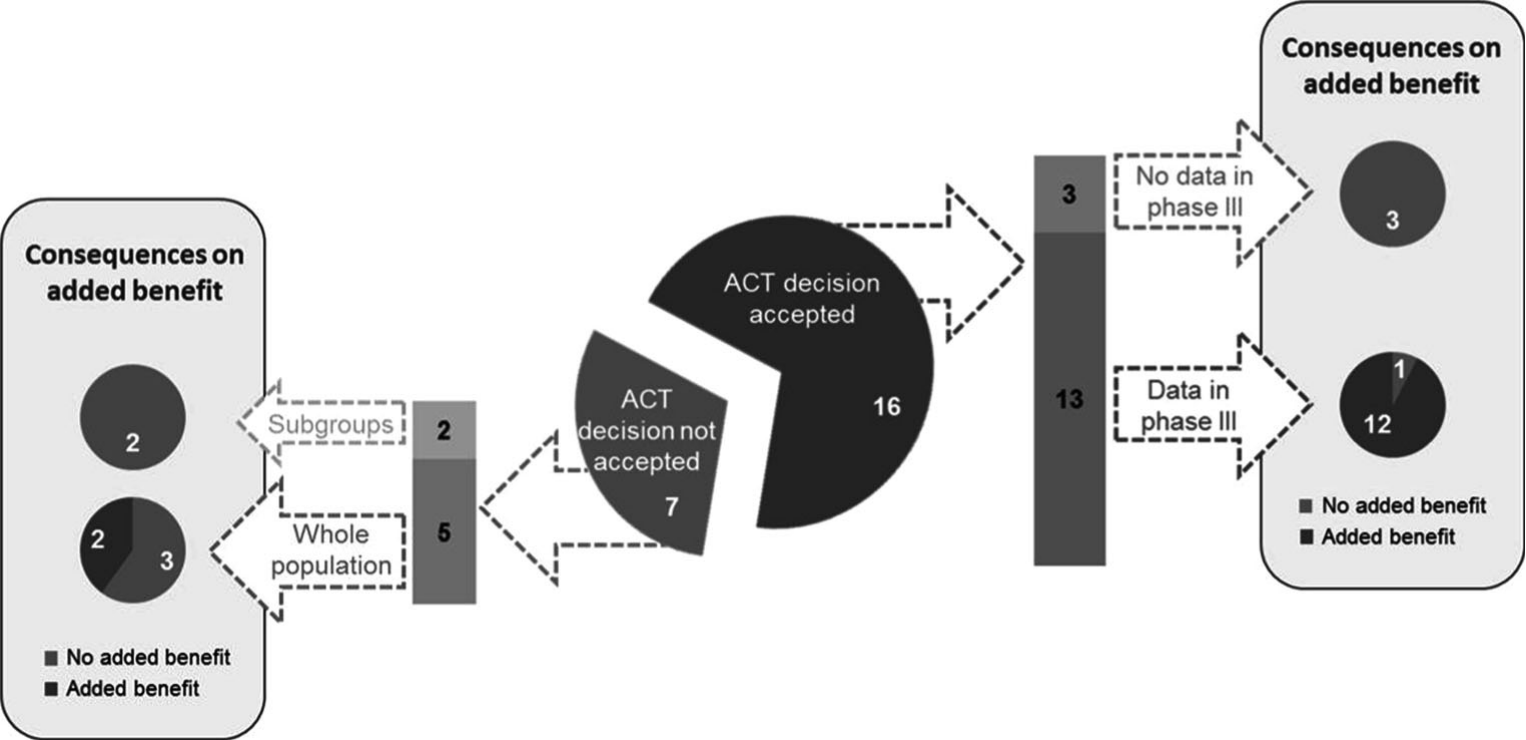
Acceptance of ACT selected by G-BA and consequences on the added benefit decision

The manufacturer used the ACT suggested by G-BA in 16 of 23 benefit dossiers (Fig. 3) [7]. The assessments of azilsartan medoxomil, bromfenac, pitavastatin and regadenoson were excluded from this analysis due to missing dossiers. In three of the 16 cases (abiraterone acetate, fampridine and fingolimod), data for the suggested comparators (e.g. for subgroups) were not available from phase III clinical trials or were not reported in the dossier (Table 4). For the remaining seven EBAs, the manufacturer did not follow the G-BA recommendations and developed dossiers based on a different ACT for all patients (cabazitaxel, aliskiren/amlodipine, linagliptin, pirfenidone and retigabine) or for specific subgroups (microbial collagenase and ticagrelor) (Table 4; Fig. 3) [7].

The ACT suggested by G-BA differed in 12 assessments from the comparator used in phase III clinical studies. Disagreement regarding the ACT was one of the major reasons for a no additional benefit conclusion by G-BA, either for the EBA as a whole or for specific subgroups within the dossier.

Due to missing data on the suggested comparator, G-BA decided that there was no additional benefit in certain subgroups in five of the assessments (abiraterone acetate, cabazitaxel, ticagrelor, fingolimod and microbial collagenase) (Table 4). Disagreement between the manufacturer and G-BA regarding the ACT was found in four EBAs (aliskiren/amlodipine, linagliptin, pirfenidone and retigabine) (Table 4) [7].

G-BA defined an individual optimised standard therapy (OST) as ACT in nine EBAs, and best supportive care (BSC) was suggested as an appropriate comparator in five assessments. Although both the manufacturer and G-BA used OST as ACT in the EBA of belimumab, the definition of OST differed between the manufacturer, IQWiG and G-BA. Whereas the studies presented by the manufacturer were not accepted by IQWiG, G-BA decided to accept inclusion of the studies and concluded a significant additional benefit for belimumab [7]. Similarly, the interpretation of OST differed between IQWiG and G-BA for the extract of *Cannabis sativa* [7].

In the EBA of fampridine, physiotherapy was used as ACT. The manufacturer’s dossier showed methodological deficits due to inadequate documentation of physiotherapy and medical interventions to the level specified by G-BA. Additionally, the study population that was used for indirect comparison included patients with lower degrees of disability than the values required for treatment with fampridine. Therefore, IQWiG and G-BA concluded that the evidence for an additional benefit was not adequate [7].

Direct comparisons between investigated drugs and ACTs are favoured by IQWiG and G-BA [4, 8]. In six of the 12 cases where the comparator used in the phase III trials differed from the ACT recommended by G-BA, the manufacturer included an indirect comparison of data from two separate trials in the dossier. Minor acceptance of these indirect comparisons by IQWiG and G-BA was evident in the evaluation of abiraterone acetate, fampridine and microbial collagenase. The indirect comparison for ticagrelor based on the major PLATO and TRITON studies was successful [7].

### Patient-relevant endpoints and benefit domains

Table 5 summarises the manufacturers’ conclusions, the IQWiG assessment, and the G-BA decisions regarding patient-relevant outcomes. Data presented by the manufacturer for mortality, morbidity and HRQoL were accepted for evaluation by IQWiG and G-BA in 11, 14 and 10 EBAs, respectively. An additional benefit in at least one endpoint was confirmed in 14 EBAs by IQWiG and/or G-BA.

**Table 5:** Additional benefits claimed by the manufacturer compared with those considered as addressed by IQWiG and G-BA

	Manufacturer^a,b^			IQWiG/G-BA^a,b^		
	Mortality	Morbidity	HRQoL	Mortality	Morbidity	HRQoL
Abiraterone acetate	+	+	+	+	+	
Aliskiren/amlodipine	+	+				
Apixaban	±	Embolism: ± DVT: +		±	Embolism: *Knee:* – *Hip:* ± DVT: +	
Belatacept	±	+	±	±	IQWiG: ± G-BA: +	±
Belimumab		+	+		G-BA: +	G-BA: ±
Boceprevir		+		±	+	
Cabazitaxel	+	+	±	+	±	
Emtricitabine/rilpivirine/tenofovir disoproxil	±	±	+			
Eribulin	+			+		
Extract of *Cannabis sativa*		+	+		G-BA: +	G-BA: ±
Fampridine		+				
Fingolimod		+	±		±	±
Ipilimumab	+	±	±	+		±
Linagliptin		±				
Microbial collagenase		+				
Pirfenidone	±	+	±	±	±	±
Retigabine		±				
Rilpivirine		+	±		IQWiG: + G-BA: ±	±
Tafamidis meglumine		+	+	±	+	±
Telaprevir		+	+		+	±
Ticagrelor	+	+		+	+	
Vandetanib		+	±			
Vemurafenib	+	±	±	+	±	±

*DVT* deep vein thrombosis

+ Additional benefit confirmed; ± No significant differences observed, no additional benefit; −Less benefit

^a^Information on http://www.g-ba.de/informationen/nutzenbewertung/ in manufacturers’ dossier and G-BA decision

^b^In case of different subgroups within an EBA, the most positive assessment is stated

In six EBAs, an additional benefit on mortality was confirmed by IQWiG and G-BA. All but one of the oncology submissions (vandetanib) were able to demonstrate survival benefit [7]. In the dossiers for abiraterone acetate, cabazitaxel, eribulin, ipilimumab and vemurafenib, an additional benefit in overall survival was reported [7]. Furthermore, ticagrelor showed both an additional overall and cardiovascular mortality benefit [7].

An additional benefit on morbidity was reported in seven IQWiG evaluations. G-BA agreed with these seven decisions and, in addition, concluded that belatacept, belimumab and the extract of *Cannabis sativa* had additional benefits on morbidity [7].

No significant differences between any of the investigated drugs and the ACT regarding HRQoL were evident and therefore no proof for additional benefit was recognised by IQWiG and G-BA for this dimension [7].

Discrepancies were observed in the interpretation of the value of surrogate endpoints reported in the EBAs of belatacept, boceprevir, telaprevir and HIV products [7]. The manufacturer of belatacept reported the glomerular filtration rate (GFR) as a surrogate endpoint for graft function; however, IQWiG did not consider GFR to be a patient-relevant endpoint, stating that it needs further validation in order to be accepted. G-BA agreed with the manufacturer and acknowledged GFR as a relevant endpoint. In hepatitis C, sustained virological response (SVR) was considered by IQWiG as a valid surrogate endpoint only for hepatocellular carcinoma. Nevertheless, G-BA accepted SVR as an overall patient-relevant endpoint in the decisions for boceprevir and telaprevir [7].

### Assessment and weighting of adverse events

**Fig. 4:**
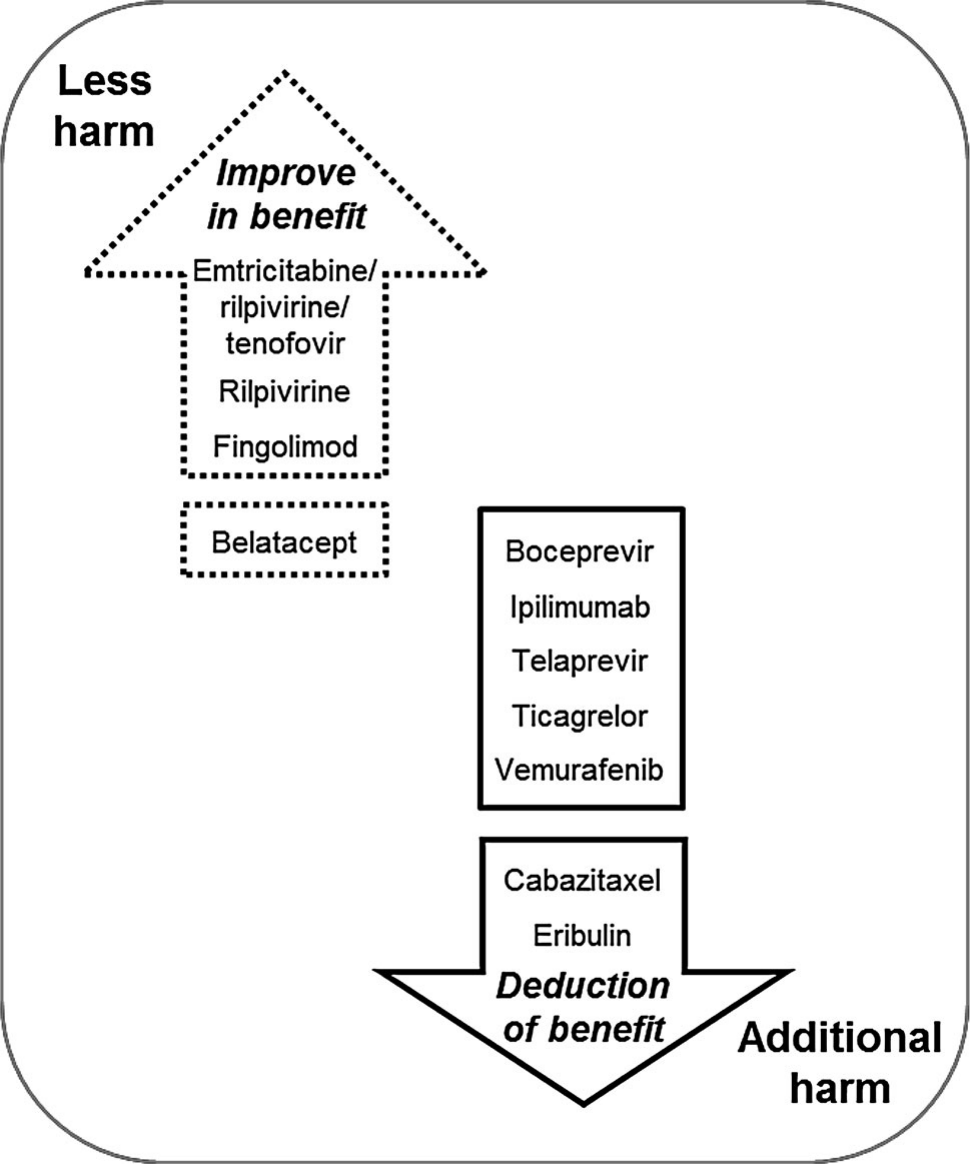
Evaluation of negative patient-relevant outcomes by G-BA in the included EBAs. For products at the end of the *arrows* this evaluation leads to an up- and downgrade, respectively. This evaluation does not influence the benefit level of the products before the gap

The EBA dossier submissions have to include all relevant safety data. Reduction of adverse events in comparison with the ACT is considered an additional benefit of a new medicine. Figure 4 shows the G-BA’s view on the adverse events of the new drugs. In seven EBAs, there was evidence of an increased number or greater severity of adverse events, thus causing a greater negative outcome on the patient compared with the ACT. Improvement in adverse events compared with the ACT was found in four EBAs. G-BA concluded that there was no proof or indication for additional harm of the new medicine in comparison with the ACT in five EBAs.

In seven EBAs (boceprevir, cabazitaxel, eribulin, ipilimumab, telaprevir, ticagrelor and vemurafenib), positive effects on patient-relevant endpoints and negative effects from side effects were considered by IQWiG to balance each other out. However, only in the cases of cabazitaxel and eribulin did G-BA follow IQWiG’s recommendation and reduce the total benefit score due to adverse events (in addition, G-BA did not agree with IQWiG on the effectiveness of cabazitaxel) [7] (Fig. 4).

The positive side-effect profile of emtricitabine/rilpivirine/tenofovir disoproxil, i.e. fewer dermatological and neurological adverse events, led to an upgrading of the overall benefit score by G-BA [7]. The additional benefit of rilpivirine was based entirely on the reduction of neurological adverse events [7]. Flu-like symptoms were improved by fingolimod, resulting in an elevated benefit score [7] (Fig. 4).

The conclusions of IQWiG and G-BA on adverse events were sometimes related to subgroup analyses (belatacept, boceprevir, fingolimod and telaprevir) [7]. In some cases, data for the relevant subgroup were considered as missing in the manufacturers’ dossier (eribulin and ticagrelor) [7]. Eribulin is the only drug so far to receive a score of less benefit by G-BA in a subgroup of patients. G-BA decided that in patients who can be re-exposed to treatment with taxane/anthracycline, eribulin has no additional benefit in terms of efficacy and the potential for additional harm, leading to a total score of less benefit in that subgroup [7].

## Discussion

In a recent report of the 22 new pharmaceutical products licensed in Germany in 2011, 46 % were considered to represent a significant innovation in terms of having a novel mechanism of action and targeting a new indication [9]. An additional 36 % of the products were considered to be partially innovative in that they provided a clinical benefit in a given indication. For 18 % of the new drugs, no real innovation could be observed (so-called me-too products) [9]. Similarly, it has been reported that around 25 % of new medicines authorised in Europe between 1995 and 2010 showed an important degree of therapeutic innovation [10–12]. The definition of an innovative medicine according to AMNOG includes additional benefit for patients in comparison with existing treatments. In a recent press release, G-BA announced that in 64 % of evaluations an additional benefit was reported [13]. Our analysis showed similar results. Out of 27 evaluations, 17 (63 %) concluded an additional benefit (Table 3). This is also in line with health technology assessments (HTA) of health authorities in other countries, such as the French Haute Autorité de Santé (HAS).

In France, the transparency committee, as part of the National Authority for Health (HAS), provides guidance on the positive listing of drugs, taking into account their comparative value and their role for the target disease. Two levels of benefit are used for each drug: the level of benefit rendered by the medicine to the patient, expressed as ‘Service Médical Rendu’ (SMR), and the level of additional benefit that the new drug is expected to provide compared with alternatives, expressed as ‘Amelioration du Service Médical Rendu’ (ASMR) in five categories from ASMR I (major improvement) to ASMR V (no improvement) [14]. The SMR determines inclusion in a positive list and the respective reimbursement rate fixed by the Association of Health Insurance Funds (UNCAM). Drugs with insufficient SMR are not recommended for reimbursement. The ASMR is taken into account when setting a price for the drug as the final outcome of the negotiations between the manufacturer and the Committee for the Pricing of Healthcare Products (CEPS).

The HAS has evaluated 22 of the 27 medicines included in our review [15]. Both the HAS and G-BA reported no additional benefit for five of these 22 common products at the same time (bromfenac, fampridine, linagliptin, microbial collagenase, retigabine). In 16 of 22 decisions, the agreement between the assessments in Germany and France was either moderate or strong (Table 6). In three cases there was a definite disagreement, whereas in one of the two health systems no additional benefit at all was assigned (combination of emtricitabine/rilpivirine/tenofovir, rilpivirine, vandetanib). This applies to both Germany and France. For example, in the case of vandetanib, an ASMR IV was assigned in France whereas in Germany, G-BA decided that there is no additional benefit. For rilpivirine monotherapy or triple-combination the opposite occurred (Table 6). Comparison with NICE was not conducted due to methodological differences between the two HTA approaches.

**Table 6:** Comparison of the assessment of common new drugs in Germany and France

Drug	G-BA assessment^a^	HAS assessment^b^	(Sub) population^c^	Agreement
Abiraterone acetate	Ind. significant (II)	Moderate ASMR III	G-BA: BSC patients	+
Apixaban	Ind. marginal (III)	Minor ASMR IV	G-BA: HIP operation	+ +
Belatacept	Ind. marginal (III)	Minor ASMR IV	HAS: young, EB-virus	++
Belimumab	Ind. significant (II)	Minor ASMR IV		−
Boceprevir	Ind. not quant. (IV)	Moderate ASMR III	HAS: ther.-experienced	+
Bromfenac	No (V)^d^	No improv ASMR V		+ +
Cabazitaxel	Ind. marginal (III)	Minor ASMR IV	HAS: 2nd line after Dtx^e^ G-BA: no Dtx re-therapy	++
Emtricitabine/rilpivirine/tenofovir	Proof marginal (III)	No improv ASMR V		− −
Eribulin	Hint marginal (III)	Minor ASMR IV		+ +
Fampridine	No (V)	No improv ASMR V		+ +
Fingolimod	Hint marginal (III)	Minor ASMR IV	G-BA: RRMS patients	++
Ipilimumab	Ind. significant (II)	Minor ASMR IV		−
Linagliptin*	No (V)^d,f,g^	No improv ASMR V	HAS: combin. therapy	+ +
Microbial collagenase	No (V)^g^	No SMR^h^		(+)
Pirfenidone	Not quant (IV)^i^	Minor ASMR IV		+
Retigabine	No (V)^d,g^	No improv ASMR V	HAS: 2line	+
Rilpivirine	Proof marginal (III)	No improv ASMR V		− −
Tafamidis meglumine	Marginal (III)^i^	Minor ASMR IV		+ +
Telaprevir	Ind. not quant (IV)	Moderate ASMR III	HAS: ther.-experienced	+
Ticagrelor	Proof signific. (II)	Minor ASMR IV	G-BA: UA/NSTEMI	−
Vandetanib	No (V)^d^	Minor ASMR IV		− −
Vemurafenib	Ind. Significant (II)	Moderate ASMR III		+

+/++/− /−−: moderate agreement/strong agreement/at least two classes difference/different direction of (additional) benefit

* Linagliptin has been compared in Germany versus sulfonylurea, in France versus sitagliptin. G-BA is now reassessing linagliptin

^a^According to the German classification scheme: major (I), significant (II), marginal (III), not quantifiable (IV), no additional benefit (V) and less benefit with proof, indication and hint as conclusion categories

^b^According to the French classification system for additional benefit ‘Amelioration du Service Médical Rendu’ (ASMR) of the Haute Autorité de Santé (HAS): ASMR I major, ASMR II important, ASMR III moderate, ASMR IV minor and ASMR V no improvement

^c^In case of more subpopulations the comparison is referring to the respective subpopulation with the highest additional benefit classification

^d^No benefit assessment dossier was submitted or no additional benefit was assigned due to formal reasons (e.g. inappropriate comparator)

^e^Docetaxel including chemotherapy

^f^Re-assessment according to §35a (5b) German Social Code Book V has started on 01.09.2012

^g^Opt-out in Germany

^h^‘Service Médical Rendu’ is referring only to the benefit, not to the additional benefit

^i^Orphan drug

**Fig. 5:**
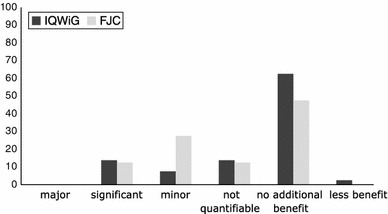
Level of additional benefit (%) in 40 assessed patient subgroups in the 23 evaluated EBAs (23 of 31 drugs, 4 drugs exempted and 4 without submitted dossier)

**Fig. 6:**
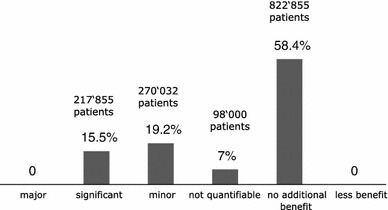
Level of additional benefit compared to % and total number of patients in 39 (Rilpivirine and Emtricitabine/Rilpivirine/Tenofovir disoproxil are referring to the same population) assessed patient subgroups in the 23 (23 from 31 drugs, 4 exempted drugs and 4 drugs without submitted dossier) evaluated EBAs. Total (sum of mean values from G-BA decisions: 1,408,742 patients) (Without the Linagliptin population of 1,219,500 patients due to the dossier re-submission)

In addition, some further aspects have to be taken into account when interpreting the results. G-BA often ‘sliced’ the total patient population into subgroups and assigned different additional benefit scores for the identified subgroups. Of the total number of 40 subgroups, an additional benefit was reported in only about 50 % (Fig. 5). The number of patients studied in each of the subgroups can be calculated from the manufacturers’ dossiers; only 40 % of the patients were in subgroups in which the new drug had an additional benefit according to the G-BA (Fig. 6).

Whereas IQWiG uses a threshold concept to define the impact that leads to a major, significant or marginal additional benefit [16], G-BA did not use this concept for its decisions. It should be mentioned that G-BA has not assigned the category of major additional benefit to any single drug evaluated so far, and it is therefore unclear what impact a drug would need to have in order for G-BA to consider it as having a major additional benefit. The pivotal trial of vemurafenib, which is indicated for the treatment of melanoma, was terminated early at the request of the regulatory bodies due to the observed improvement in overall survival benefit. In contrast to the manufacturer, the G-BA did not consider that the statement by the regulatory bodies should be interpreted as proof of a major additional benefit [17].

This analysis indicates disagreement between the ACTs suggested by G-BA and the manufacturers (Table 4). Ten of 23 EBAs (44 %) showed only limited agreement (6 EBAs) or disagreement (4 EBAs) in the selection of ACT. Three manufacturers applied the comparator that had been suggested by G-BA even though relevant data were not available. As a consequence, these three products received negative G-BA decisions due to the lack of data.

Until now, the advice meetings of the manufacturers with G-BA have taken place at a late stage in the clinical development process, after initiation of phase III studies; therefore the design of the phase III studies could not take into account the feedback and requests of G-BA. The most recent legislation requires the involvement of the health authorities during early advice meetings (before initiation of phase III clinical trials) between the pharmaceutical manufacturer and G-BA. It is hoped that discrepancies concerning the comparators used for phase III clinical studies and benefit assessments, as well as discrepancies regarding the selection of patient-relevant endpoints, will diminish over time as a result of this initiative.

Many of the ACTs suggested by G-BA lack appropriate clinical evidence. When applying the IQWiG hierarchy of evidence, many of the suggested ACTs would not even qualify for a hint, rendering the comparison of the innovative treatment with the ACT somewhat arbitrary. The most frequently applied ACT was best supportive care (BSC). However, the definition of BSC varied considerably. For example, physiotherapy was selected as the comparator for the multiple sclerosis drug fampridine [7] despite a lack of evidence from clinical trials that physiotherapy offers a statistically significant benefit. In the assessment of cabazitaxel for the treatment of prostate cancer, the manufacturer used mitoxantrone as ACT, because it is used (with or without prednisone/prednisolone) in over 50 % of patients in Germany. In contrast, G-BA selected dexamethasone, prednisone, prednisolone or methylprednisolone, in addition to BSC as the ACT, and considered mitoxantrone to be part of BSC [7]. Similarly, percutaneous needle fasciotomy was chosen by G-BA as the ACT for microbial collagenase in two subgroups within the dossier [7], even though percutaneous needle fasciotomy (PNF) is used in less than 10 % of cases in general practice in Germany and there are no randomised clinical trial (RCT) data regarding PNF’s effect on patient-relevant outcomes.

There is also some debate over which endpoints should be considered to be relevant for patients. The endpoints in clinical trials differ across disease areas. For example, in oncology there is a focus on survival benefit and measures of disease morbidity, such as progression-free or disease-free survival, while in virology, endpoints primarily address viral load and in rheumatology, clinical composite scores are the current standard. While these endpoints have become established and recognised by the regulatory bodies, they are not necessarily accepted by HTA bodies. For example, PFS was not considered a relevant endpoint in the assessment of various oncology drugs (e.g. abiraterone acetate, cabazitaxel and vemurafenib) and SVR was not considered relevant by IQWiG in the assessment of boceprevir and telaprevir for the treatment of hepatitis C. However, such endpoints may be very valuable in detecting changes in morbidity before they become symptomatic for patients (i.e. before functional deficits occur). Furthermore, progression of cancer usually requires a change in therapy and is often associated with anxiety for patients, suggesting that PFS should be considered a patient-relevant endpoint. Similarly, SVR is a marker of hepatitis C progression that may detect changes in disease severity, even before they become symptomatic. Although IQWiG considered SVR irrelevant, G-BA decided to accept SVR as a patient-relevant endpoint, demonstrating inconsistency in the interpretation for patient relevance.

The German Social Law discriminates three dimensions of patient-relevant endpoints: mortality, morbidity and HRQoL [6]. However, established disease-specific endpoints in clinical trials do not address the impact on all three dimensions [18]. In particular, none of the additional benefits granted by the G-BA were based on HRQoL, which indicates major methodological challenges with the reliable assessment of HRQoL within clinical development programmes.

As described in the EMA benefit-risk programme [19], regulatory bodies put enormous emphasis on the appropriate classification of adverse events and on the balancing of risks and benefits. As the impact on adverse events is a key component of the G-BA decisions on overall additional benefit, some issues require further clarification. The basis of the EMA grading of adverse events is the Common Terminology Criteria for Adverse Events (CTCAE) classification [20]. Within CTCAE investigations, symptomatic myocardial infarction, pure abnormalities of cardiac enzymes, and a decrease in white blood cells to <1,000 mm^3^ qualify as grade 4 adverse events. It may be assumed that G-BA would not consider a transient decrease in white blood cell count as patient relevant, whereas it would consider a symptomatic myocardial infarction to be patient relevant. A systematic approach to classify the severity of patient-relevant adverse events is therefore required. The G-BA has to further define and clarify its categorisation of adverse events [12] beyond the definition used by the EMA.

The evidence hierarchy within the AMNOG process considers RCTs as the gold standard for evidence development [8]. It should be noted, however, that clinical trials are usually powered for efficacy but not for adverse events. Therefore, alternative methods of evidence generation have to be developed and applied that take into account clinical aspects, such as manageability and reversibility of adverse events.

Balancing benefit and risk is a key feature of the EMA review process. Granting marketing authorisation to a drug implies a positive benefit-risk balance. In contrast, a G-BA decision of less benefit (as occurred in one of the subgroups in the assessment of eribulin) implies a negative benefit-risk balance. An alignment of regulatory and G-BA approaches therefore seems critical to provide patients with a clear understanding of the benefit-risk ratio.

Almost 2 years after introduction of the AMNOG legislation, only very limited information is available regarding the full AMNOG process, e.g. details of price negotiation are lacking in the public domain. Nevertheless, a considerable amount of experience has been gained in the EBA procedures. There is significant variability in the additional benefit reported between pharmaceutical manufacturers, IQWiG and G-BA, and this becomes apparent in four key areas: ACT selection, subgroup definition, definition of patient-relevant endpoints (mortality, morbidity and HRQoL), and the impact of selected adverse events on benefit assessment. It also remains unclear under what circumstances a manufacturer may deviate from the assessment methodologies of IQWiG and G-BA, and to what extent benefit assessment will be penalised for such deviations. The hope remains, however, that increased experience [21] and earlier interaction between manufacturers, regulatory authorities, and HTA authorities, may encourage more streamlined and integrated regulatory and HTA programmes.
